# Identification of antibody-drug conjugate payloads that are substrates of ATP-binding cassette drug efflux transporters

**DOI:** 10.20517/cdr.2025.151

**Published:** 2026-01-12

**Authors:** Jacob S. Roth, Hui Guo, Lu Chen, Min Shen, Omotola Gbadegesin, Robert W. Robey, Michael M. Gottesman, Matthew D. Hall

**Affiliations:** ^1^National Center for Advancing Translational Sciences, National Institutes of Health, Rockville, MD 20850, USA.; ^2^Laboratory of Cell Biology, National Cancer Institute, National Institutes of Health, Bethesda, MD 20892, USA.

**Keywords:** ABC transporter, P-glycoprotein, ABCG2, antibody-drug conjugate, drug resistance

## Abstract

**Aim:** Antibody-drug conjugates (ADCs) feature an antibody recognizing a specific protein joined to a potent toxic payload. Numerous ADCs have received U.S. Food and Drug Administration (FDA) approval; however, clinical resistance arises. Resistance mechanisms include decreased expression or mutation of the antibody target, impaired payload release, or increased expression of adenosine triphosphate (ATP)-binding cassette (ABC) efflux transporters associated with multidrug resistance. We therefore sought to characterize the interactions of ABC multidrug transporters with ADC payloads.

**Methods:** We performed a high-throughput screen with 27 common ADC payloads using cell lines expressing ABC transporters P-glycoprotein [P-gp, encoded by ABC subfamily B member 1 (*ABCB1*)] or ABC subfamily B member G2 (ABCG2, encoded by *ABCG2*). Confirmatory assays were also performed using cells transfected to express P-gp, ABCG2, or multidrug resistance-associated protein 1 (MRP1, encoded by *ABCC1*).

**Results:** Several commonly used ADC payloads were substrates of P-gp, including calicheamicin γ1, monomethyl auristatin E, mertansine (DM1), and ravtansine (DM4). All the pyrrolobenzodiazepines tested - SJG136, SGD-1882, SG2057, and SG3199 - were substrates of P-gp, ABCG2, and MRP1. The modified anthracyclines nemorubicin and its metabolite PNU-159682 were poorly transported by both ABCB1 and ABCG2 and displayed nanomolar to picomolar toxicity. Further, we found that the efficacy of the FDA-approved ADC mirvetuximab soravtansine, with DM4 as the toxic payload, was decreased in cell lines expressing P-gp. In contrast, Duocarmycin DM and PNU-159682 were exquisitely toxic to a panel of 99 cancer cell lines of varying origins.

**Conclusion:** Several commonly used ADC payloads can be transported by ABC transporters, potentially leading to transporter-mediated drug resistance in patients. Future ADCs should be developed using payloads that are not ABC transporter substrates.

## INTRODUCTION

Antibody-drug conjugates (ADCs) have garnered significant attention as a strategy for selectively targeting tumor cells. ADCs consist of an antibody that targets a specific cell-surface antigen, conjugated to a small-molecule toxin (payload) via a cleavable linker^[1]^. Following systemic distribution and binding to the antigen, the ADC is internalized and processed within the target cell lysosomes, releasing the toxin to exert its cytotoxic effects. Thus, provided the antibody targets a protein or epitope that is selectively expressed on the extracellular surface of cancer cells and minimally on other cell types in the body, the ADC should selectively kill tumor cells^[2]^. The first ADC was granted U.S. Food and Drug Administration (FDA) approval in 2000 for the treatment of acute myeloid leukemia (AML); gemtuzumab ozogamicin targets cluster of differentiation 33 (CD33) and carries N-acetyl-calicheamicin γ1 as the cytotoxic payload^[3]^. Since then, several other ADCs have been approved for the treatment of cancer including brentuximab vedotin for Hodgkin lymphoma^[4]^, trastuzumab emtansine for breast cancer^[5]^, trastuzumab deruxtecan for breast cancer^[6]^ [recently approved for any human epidermal growth factor receptor 2 (HER2)-positive solid cancer^[7]^], loncastuximab tesirine for diffuse large B-cell lymphoma^[8]^, enfortumab vedotin for urothelial cancers^[9]^ and mirvetuximab soravtansine (MIRV) for cisplatin-resistant ovarian cancers^[10]^. FDA-approved ADCs, along with ADCs in clinical trials, have employed highly cytotoxic natural product-derived payloads that cause cell death via various modalities including induction of DNA damage, disruption of microtubules, and inhibition of topoisomerase^[11]^. Many of these agents originate from molecules that were explored as small-molecule (untargeted) chemotherapeutics, but potent cytotoxic side effects led to their abandonment. A partial listing of approved ADCs and ADCs currently in clinical trials is included in Table 1.

**Table 1:** Approved and clinical candidate ADC small-molecule payloads

**Name**	**Brand name**	**Payload**	**Linker**	**FDA status**
Gemtuzumab ozogamicin	Mylotarg	Calicheamicin	Cleavable	Approved
Inotuzumab ozogamicin	Besponsa	Calicheamicin	Cleavable	Approved
Trastuzumab deruxtecan	Enhertu	Dxd (exatecan derivative)	Cleavable	Approved
Datopotamab deruxtecan	Datroway	Dxd (exatecan derivative)	Cleavable	Approved
Ado-trastuzumab emtansine	Kadcyla	DM1 (maytansinoid analog)	Non-cleavable	Approved
Mirvetuximab soravtansine	Elahere	DM4 (maytansinoid analog)	Cleavable	Approved
Brentuximab vedotin	Adcetris	MMAE	Cleavable	Approved
Polatuzumab vedotin	Polivy	MMAE	Cleavable	Approved
Enfortumab vedotin	Padcev	MMAE	Cleavable	Approved
Tisotumab vedotin	Tivdak	MMAE	Cleavable	Approved
Telisotuzumab vedotin	Emrelis	MMAE	Cleavable	Approved
Loncastuximab tesirine	Zynlonta	SG3199 (pyrrolobenzodiazepine dimer)	Cleavable	Approved
Sacituzumab govitecan	Trodelvy	SN-38 (active metabolite of irinotecan)	Cleavable	Approved
Disitamab vedotin	N/A	MMAE	Cleavable	In clinical trials
Depatuxizumab mafodotin	N/A	MMAF	Non-cleavable	In clinical trials
Zilovertamab vedotin	N/A	MMAE	Cleavable	In clinical trials
Vadastuximab talirine	N/A	SGD-1882 (pyrrolobenzodiazepine dimer)	Cleavable	In clinical trials
Camidanlumab tesirine	N/A	SG3199 (pyrrolobenzodiazepine dimer)	Cleavable	In clinical trials
Trastuzumab duocarmazine	N/A	Duocarmycin (Seco-DUBA)	Cleavable	In clinical trials
Pivekimab sunirine	N/A	DGN549 (Indolinobenzodiazepine pseudodimer)	Cleavable	In clinical trials
Belantamab mafodotin	Blenrep	MMAF	Non-Cleavable	Withdrawn

ADC: Antibody-drug conjugate; FDA: U.S. Food and Drug Administration; DM1: mertansine; DM4: ravtansine; MMAE: monomethyl auristatin E; N/A: not available; MMAF: monomethyl auristatin F.

Despite responses of some cancers to ADCs, resistance to treatment is observed in the clinic. Clinically observed mechanisms of resistance include downregulation or mutation of the ADC antibody target, failure of lysosomes to release the payload, activation of alternative survival pathways, and the overexpression of efflux transporters^[12-16]^. Patients whose leukemic blasts had lower levels of CD33 were found to respond more poorly to treatment with gemtuzumab ozogamicin *vs.* patients with blasts expressing higher levels^[17]^. Similarly, patients whose tumors were found to express a truncated form of HER2 that does not bind to trastuzumab, p95HER2, were found to be largely resistant to trastuzumab emtansine^[18]^. Decreased payload release in patient-derived samples has been linked to drug accumulation in the lysosomes due to increased lysosomal pH^[19]^. Finally, overexpression of the adenosine triphosphate-binding cassette B1 (*ABCB1*) gene - which encodes the adenosine triphosphate (ATP)-binding cassette (ABC) transporter P-glycoprotein (P-gp) - has been inversely correlated with response to gemtuzumab ozogamicin in patients with AML^[17,20]^. While individual reports of ABC transporter-mediated resistance of individual ADCs exist^[21,22]^, no systematic examination of the relationship between toxin sensitivity and ABC transporter expression has been reported.

The objective of this study was to determine whether several commonly used ADC payloads were substrates for transport by ABC multidrug transporters with the goal of identifying cytotoxic agents that were not subject to transport out of the cell. P-gp substrates were identified by comparing compound activity between paired sets of low-expressing (sensitive) and over-expressing (resistant) cell lines for both P-gp and ABC subfamily G member 2 (ABCG2). Four paired cell sets were utilized - a drug-selected and transfected set for each transporter. Hits were confirmed in cells transfected to express P-gp, ABCG2, or multidrug resistance-associated protein 1 (MRP1, encoded by *ABCC1*). These results should aid investigators in designing ADCs that are not subject to ABC transporter-mediated resistance.

## METHODS

### Cell lines

HEK-293 cells were purchased from the American Type Culture Collection (ATCC, Manassas, VA) and transfected with empty vector (pcDNA) or vector containing the human *ABCB1* (MDR-19), *ABCG2* (R-5), or *ABCC1* gene (MRP1) and were grown in Eagle’s Minimum Essential Medium (EMEM) with 2 mg/mL Geneticin (G418) added to maintain transporter expression^[23]^. The HeLa derivative KB-3-1 was purchased from ATCC and the P-gp overexpressing KB-8-5-11 subline was generated by selection with 100 ng/mL colchicine. The lines were maintained in Dulbecco’s Modified Eagle Medium (DMEM); KB-8-5-11 additionally received 100 ng/mL colchicine^[24]^. OVCAR8, NCI-ADR-RES (doxorubicin-selected, P-gp expressing OVCAR8 cell line), and H460 parental cells were obtained from the Division of Cancer Treatment and Diagnosis Tumor Repository, National Cancer Institute at Frederick, MD, and were cultured in RPMI. The ABCG2-expressing subline H460 MX20 was generated by selecting H460 cells in 20 nM mitoxantrone^[25]^. Media for all cell lines above was supplemented with 10% fetal bovine serum (FBS) and 1x penicillin-streptomycin. We also performed screening against a panel of 99 cancer cell lines; cell line source and culture conditions are reported in Supplementary Table 1.

### High-throughput screen

We explored 27 common ADC payloads, listed in Table 2, in two orthogonal, paired cell line sets - a drug-selected and transfected set for each transporter - to identify substrates of the ABC transporters P-gp and ABCG2. To broadly assess ADC payload toxicity, we tested several cell lines of various origins that were acquired and grown in conditions reported in Supplementary Table 1. As noted in Table 2, compounds were sourced from Levena Biopharma (San Diego, CA), Selleck Chem (Houston, TX), ChemieTek (Indianapolis, IN), or Microsource Discovery Systems (Gaylordsville, CT). Screening was performed as previously described^[26]^. Briefly, cells were plated at a density of 500 cells/well in 1536-well plates, with compounds added using a 1536-head pin tool (Kalypsys, San Diego, CA). Cells were incubated with cytotoxic drugs in the presence or absence of known inhibitors of the P-gp and ABCG2 pumps, tariquidar (TQR; 1 µM) and Ko143 (5 µM) respectively. Inhibition of efflux can restore intracellular accumulation; thus, re-sensitization of resistant cells confirms cytotoxic compounds are transporter substrates. Cells were incubated with drugs at various concentrations and processed with CellTiterGlo reagent (Promega, Madison, WI) to determine viability after 48 h. P-gp substrates were identified by comparing compound activity between paired cell line sets of low (sensitive) and high (resistant) transporter expression.

**Table 2:** ADC payloads tested in the HTS

**Compound**	**Vendor**	**Mechanism of action**
α-Amanitin	Levena Biopharma	RNA polymerase inhibitor
Ansamitocin P3	Levena Biopharma	Microtubule disruptor
Auristatin E	Levena Biopharma	Microtubule disruptor
Auristatin F	Levena Biopharma	Microtubule disruptor
Calicheamicin γ1	Levena Biopharma	DNA damage
Camptothecin	Selleck Chem	Topoisomerase inhibition
Dasatinib	Selleck Chem	Tyrosine kinase inhibitor
DM1	Levena Biopharma	Microtubule disruptor
DM4	Levena Biopharma	Microtubule disruptor
Dolastatin 10	Levena Biopharma	Microtubule disruptor
Doxorubicin	Selleck Chem	Topoisomerase inhibition
Duocarmycin DM	Levena Biopharma	DNA alkylation
Duocarmycin MA	Levena Biopharma	DNA alkylation
Duocarmycin SA	Levena Biopharma	DNA alkylation
Duocarmycin TA	Levena Biopharma	DNA alkylation
Maytansinol	Levena Biopharma	Microtubule disruptor
MMAE	Levena Biopharma	Microtubule disruptor
MMAF	Levena Biopharma	Microtubule disruptor
Monomethyl dolastatin 10	Levena Biopharma	Microtubule disruptor
N-acetyl-calicheamicin γ1	Levena Biopharma	DNA damage
Nemorubicin	Levena Biopharma	Topoisomerase inhibition
PBD Dimer (SGD-1882)	Levena Biopharma	DNA damage
PNU-159682	Levena Biopharma	Topoisomerase inhibition
SN-38	ChemieTek	Topoisomerase inhibition
Tubulysin IM-2	Levena Biopharma	Microtubule disruptor
Tubulysin M	Levena Biopharma	Microtubule disruptor
Vinblastine sulfate	Microsource Discovery	Microtubule disruptor

ADC: Antibody-drug conjugate; HTS: high-throughput screen; DM1: mertansine; DM4: ravtansine; MMAE: monomethyl auristatin E; MMAF: monomethyl auristatin F.

### Confirmatory experiments on screen hits

Three-day cytotoxicity assays were performed using various payloads including other members of the pyrrolobenzodiazepine dimer (PBD) class, as well as the ADC MIRV (obtained from MedChemExpress, Monmouth Junction, NJ), with pcDNA (empty vector transfected), MDR-19 (*ABCB1* transfected), R-5 (*ABCG2* transfected) and MRP1 (*ABCC1* transfected) cells. Briefly, cells were plated (5,000 cells/well) in 96-well plates and allowed to attach overnight, after which the desired payloads or the ADC at the indicated concentrations were added and incubated with cells for three days. CellTiter Glo reagent (Promega) was then used according to the manufacturer’s directions to assess GI_50_ (half-maximal growth inhibitory concentration) values.

### Data analysis

To evaluate compound activity in the screening experiments, concentration-response curves (CRCs) were generated for each sample by plotting normalized response data against compound concentration. These curves were modeled using a four-parameter logistic regression, which provided key pharmacological parameters such as the half-maximal inhibitory concentration (IC_50_) and maximal response (efficacy)^[27]^. While many compounds showed well-defined, sigmoidal dose–response behavior with both upper and lower asymptotes, others displayed atypical or poor-quality CRCs, including shallow slopes, incomplete asymptotes, or responses derived from only a single active concentration point. Such cases were classified as low-confidence actives due to limited or ambiguous activity profiles. Furthermore, a compound’s area under the curve (AUC) - calculated based on the screening data analysis and curve fittings - served as an integrated measure of compound activity, capturing both potency and efficacy. It was utilized for direct comparison of activity outcome across different cell lines and experimental conditions^[26]^.

## RESULTS

### A high-throughput screen identifies ADC payloads as substrates of P-gp and ABCG2

We evaluated 27 common ADC payloads in two orthogonal, paired cell sets to identify substrates of the ABC transporters P-gp and ABCG2 [Table 2]. Cytotoxic payloads were tested against two pairs of cell lines, one parental and one transporter-expressing line. For P-gp, the pairs were: (1) the parental KB 3-1 human adenocarcinoma cell line and its colchicine-selected, P-gp-overexpressing sub-line KB 8-5-11; and (2) the HEK pcDNA human embryonic kidney cell line (transfected with an empty vector plasmid control) and its *ABCB1* stably transfected sub-line MDR-19. For ABCG2, the pairs were: (1) the parental H460 human lung carcinoma cell line and its mitoxantrone-selected, ABCG2-overexpressing sub-line H460 MX20; and (2) the same HEK pcDNA parent cell line as a comparator for the *ABCG2*-transfected HEK cell line, R-5. Transporter-specific efflux was demonstrated by testing all toxins for sensitization of transporter-expressing cells in the presence of either the P-gp inhibitor TQR or the ABCG2 inhibitor Ko143. As such, six cell line conditions were used to evaluate toxins as P-gp substrates, and six cell line conditions were also used for evaluation of ABCG2 substrates.

**Figure 1:**
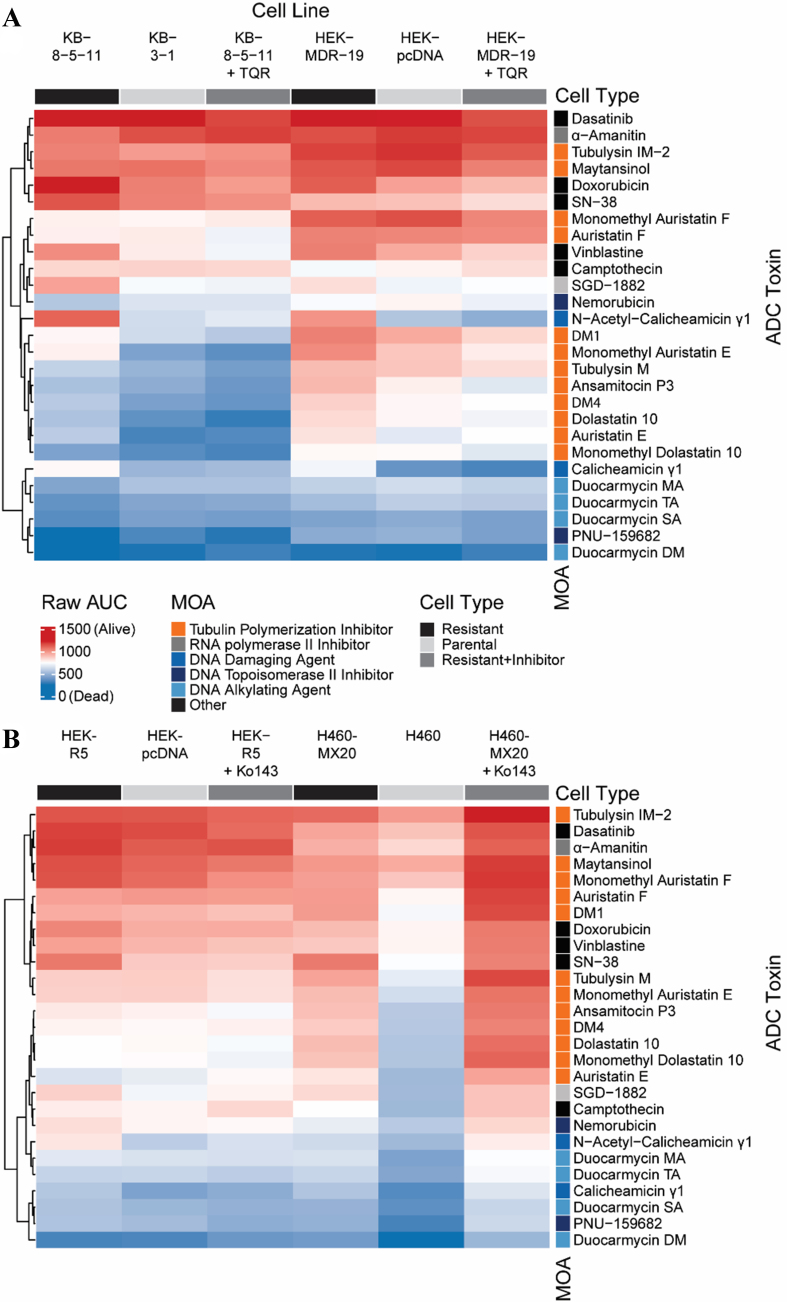
Heatmap of ADC payload activity across multidrug-resistant and control cell lines. (A) P-gp efflux potential: Unsupervised clustering of AUC values from dose-response viability assays of a panel of cytotoxic agents tested against parental, multidrug-resistant, and genetically modified KB and MDR-19 cell lines. Cell lines include KB-3-1 (parental) and KB-8-5-11 (P-gp overexpressing); HEK-293-derived MDR-19 (*ABCB1* transfected) and its vector control (pcDNA), as well as P-gp–inhibited counterparts (+TQR, tariquidar); (B) ABCG2 efflux potential: Unsupervised clustering of AUC values from dose-response viability assays of a panel of cytotoxic agents tested against parental, genetically modified, and multidrug-resistant HEK and H460 cell lines. Cell lines include H460 (parental) and H460-MX20 (ABCG2 overexpressing); HEK-derived HEK-R5 (ABCG2 transfected) and its vector control (pcDNA), as well as ABCG2–inhibited counterparts (+Ko143). ADC: Antibody-drug conjugate; P-gp: P-glycoprotein; AUC: area under the curve; ABCB1: adenosine triphosphate-binding cassette B1; TQR: tariquidar; MOA: mechanism of action.

AUC values were calculated for each cell line with all payloads in the presence or absence of a specific inhibitor as previously described^[26]^ and are displayed in the heat maps in Figure 1. In the heatmaps, red denotes high cell death and thus a more potent compound, while blue denotes less cell death. In Figure 1A, cells expressing P-gp, when compared to parental cells, were found to be less sensitive (i.e., resistant) to several ADC payloads, including calicheamicin γ1, dolostatin 10, monomethyl auristatin E (MMAE), tubulysin M, mertansine (DM1), and ravtansine (DM4). When the P-gp positive lines were incubated with the inhibitor TQR, resistance to calicheamicin γ1, dolostatin 10, MMAE, tubulysin M, DM1 and DM4 was reversed. In the Figure 1A heat map, grouping the parental cell line next to the resistant cells plus efflux inhibitor highlights that the KB 8-5-11 line treated with TQR exhibited sensitivities similar to the parental KB-3-1 line; the MDR-19 line with TQR resembled the pcDNA-transfected cells, whereas the P-gp-expressing cells showed a distinctly different response. The cytotoxicity of nemorubicin, its metabolite PNU-159682 and the duocarmycin compounds appeared to be unaffected by P-gp levels.

As shown in Figure 1B, few compounds appeared to be substrates of ABCG2, with the known substrate SN-38 being the most prominent compound that was subject to transport. Addition of the ABCG2 inhibitor Ko143 did not appear to re-sensitize the resistant cells, as R-5 cells with Ko143 did not resemble the sensitivity of pcDNA-transfected cells, and H460 MX20 cells with Ko143 responded similarly to H460 MX20 cells, rather than with the corresponding parental cell line. In line with efflux by ABCG2, the addition of Ko143 reversed resistance to SN-38.

To cross-validate efflux by the transporter of interest, we calculated and compared differences in AUC (deltaAUC) values between the parental and resistant lines and between the resistant lines in the absence or presence of inhibitor, as previously described [Supplementary Figure 1A]^[26]^. Comparing deltaAUC values between the pcDNA/MDR-19 and MDR-19 + TQR pairs and the KB 3-1/KB 8-5-11 and KB 8-5-11 + TQR pairs, we found good correlation, with r^2^ values of 0.72 and 0.92, respectively [Supplementary Figure 1B]. The results with P-gp contrasted with those of ABCG2, as few compounds were identified as ABCG2 substrates; the deltaAUC values for the pcDNA/R-5 and R-5 + Ko143/R-5 pairs and the H460/H460MX20 and H460 + Ko143/H460MX20 pairs showed poor correlation, with r^2^ values of 0.32 and 0.31, respectively [Supplementary Figure 1C]. Thus, more payloads appeared to be transported by P-gp than ABCG2.

### Validation of high-throughput screen hits

**Figure 2:**
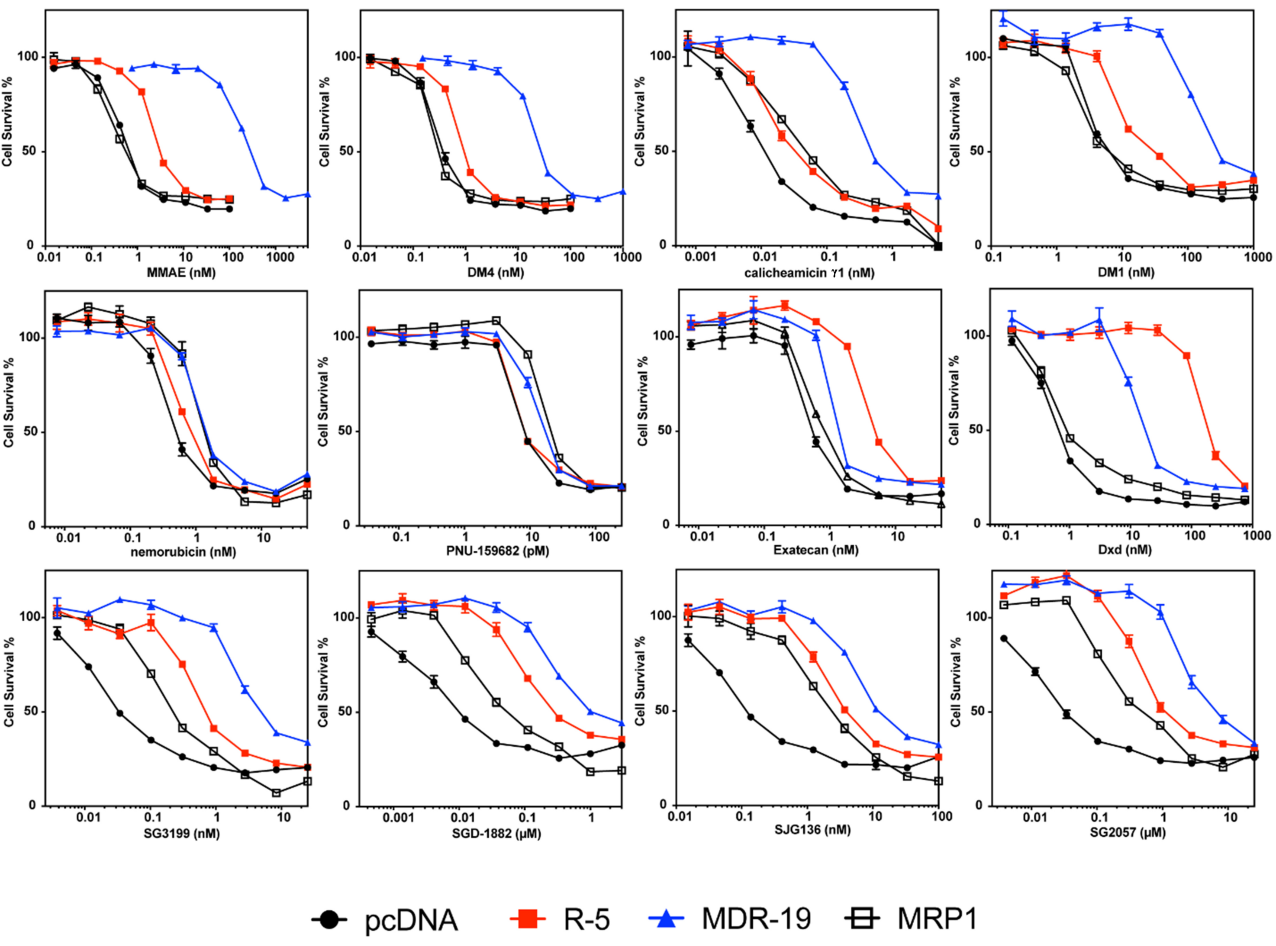
Confirmatory cytotoxicity assays with screen hits and additional ADC payloads. Three-day cytotoxicity assays were performed with the noted compounds as described in the Materials and Methods using HEK-293 cells transfected with empty vector (pcDNA, black dot), or vectors containing *ABCG2* (R5, red square), *ABCB1* (MDR-19, blue triangle), or *ABCC1* (MRP1, black box). Results from one of three independent experiments are shown. Cytotoxicity data are summarized in Table 3. ADC: Antibody-drug conjugate; ABCG2: ABC subfamily G member 2; ABCB1: adenosine triphosphate-binding cassette B1; MRP1: multidrug resistance-associated protein 1; MMAE: monomethyl auristatin E; DM4: ravtansine; DM1: mertansine.

We validated selected screening results, adding a cell line (MRP1) developed by transfecting HEK293 cells with a plasmid containing the *ABCC1* gene [Figure 2 and Table 3]. Consistent with data from the initial screen, MMAE, DM4, calicheamicin γ1, and DM1 were confirmed as avid substrates of P-gp and we noted a small degree of resistance conferred to these payloads by ABCG2 [Figure 2]. Monomethyl auristatin F (MMAF) was not a substrate of any of the transporters, although it was less potent than the related compound MMAE [Table 3]. We also confirmed that nemorubicin and PNU-159682 were not substrates of any of the transporters examined, making these better ADC payloads; PNU-159682 is exquisitely toxic, with GI_50_ values in the picomolar range [Table 3].

**Table 3:** Cross-resistance profile to select ADC payloads in cells expressing P-gp, ABCG2 or MRP1

		**ABCB1**		**ABCG2**		**ABCC1**	
**Drug**	**pcDNA**	**MDR19**	**RR**	**R5**	**RR**	**MRP1**	**RR**
DM1 (nM)	7.8 ± 1.4	410 ± 120	52	35 ± 4.1	4.5	9.5 ± 3.7	1.2
DM4 (nM)	0.43 ± 0.075	42 ± 20	96	1.4 ± 0.49	3.3	0.58 ± 0.37	1.3
MMAE (nM)	0.90 ± 0.23	360 ± 110	400	5.1 ± 1.9	5.7	1.2 ± 0.80	1.3
MMAF (μM)	0.99 ± 0.097	2.0 ± 1.7	2.0	0.99 ± 0.097	1.0	1.0 ± 0.39	1.0
Calicheamicin γ1 (nM)	0.010 ± 0.0042	0.73 ± 0.41	71	0.029 ± 0.0047	2.9	0.028 ± 0.017	2.7
Exatecan (nM)	0.59 ± 0.19	1.4 ± 0.15	2.4	5.0 ± 0.80	8.5	1.0 ± 0.19	1.8
Deruxtecan (nM)	0.82 ± 0.18	17 ± 1.2	20	190 ± 29	240	1.1 ± 0.40	1.4
Nemorubicin (nM)	0.76 ± 0.31	1.5 ± 0.15	2.0	1.1 ± 0.27	1.4	1.6 ± 0.21	2.2
PNU-159682 (pM)	13 ± 9.6	16 ± 3.7	1.2	13 ± 8.4	1.0	19 ± 8.6	1.4
SJG136 (nM)	0.098 ± 0.019	16 ± 6.2	170	4.6 ± 0.68	47	1.2 ± 1.1	12
SGD-1882 (nM)	0.0066 ± 0.0028	0.85 ± 0.34	130	0.22 ± 0.093	33	0.095 ± 0.036	14
SG2057 (nM)	0.025 ± 0.0087	5.7 ± 1.2	230	0.73 ± 0.34	29	0.27 ± 0.20	11
SG3199 (nM)	0.045 ± 0.011	5.6 ± 1.6	120	0.90 ± 0.31	20	0.21 ± 0.026	4.6

Results are mean GI_50_ values +/- standard deviation. The RR value was computed by dividing the GI_50_ value for the lines expressing the ABC transporters by the empty vector (pcDNA) line. Results are from three independent experiments. ADC: Antibody-drug conjugate; P-gp: P-glycoprotein; ABCG2: ABC subfamily G member 2; MRP1: multidrug resistance-associated protein 1; ABCB1: adenosine triphosphate-binding cassette B1; RR: relative resistance; DM1: mertansine; DM4: ravtansine; MMAE: monomethyl auristatin E; MMAF: monomethyl auristatin F; ABC: adenosine triphosphate (ATP)-binding cassette.

Overexpression of ABCG2 conferred about 10-fold resistance to the camptothecin derivative exatecan, much less than the 96-fold resistance for SN-38 we previously reported for ABCG2-transfected cells^[28]^. This is consistent with previous reports demonstrating that ABCG2 confers less resistance to exatecan compared to other camptothecin derivatives that are ABCG2 substrates^[29]^. However, the exatecan derivative Dxd (deruxtecan) was readily transported by ABCG2 and was also a substrate for P-gp [Figure 2 and Table 3].

As our screen originally contained only one member of the PBD dimer family (SGD-1882), we expanded the number of PBD dimer payloads examined. We found that HEK293 cells expressing any of the three transporters conferred some resistance to all PBD dimers examined: SG3199, SGD-1882, SJG136 (also known as SG2000), and SG2057. P-gp overexpression was found to confer the highest levels of resistance, followed by ABCG2 and finally MRP1 [Figure 2 and Table 3].

### P-gp overexpression confers resistance to MIRV

**Figure 3:**
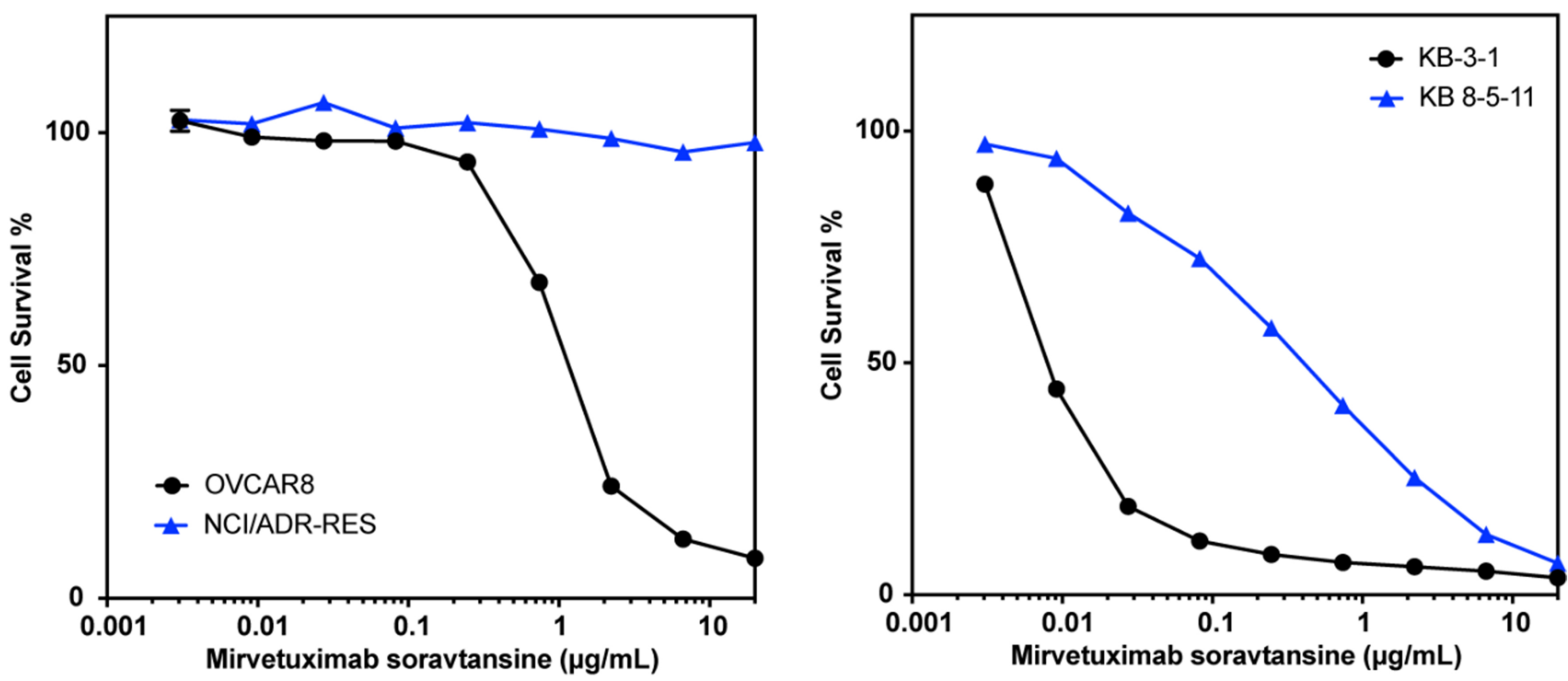
P-gp overexpression confers resistance to treatment with MIRV. Three-day cytotoxicity assays were performed with MIRV as described in the Materials and Methods using the OVCAR8/NCI-ADR-RES pair (left) or the KB 3-1/KB 8-5-11 pair (right). Results from one of two independent experiments are shown. P-gp: P-glycoprotein; MIRV: mirvetuximab soravtansine.

The screening approach described here evaluated small-molecule cytotoxic drugs employed as payloads in experimental and therapeutic ADCs. Examining how drug transporter-mediated susceptibility confers resistance to an ADC (where the toxin is liberated from the antibody within the cell and subsequently effluxed) is important for extrapolating our results. We thus tested the effect of P-gp expression on the sensitivity of cultured cells to an ADC. MIRV is an ADC that was approved by the FDA in 2022 for the treatment of folate receptor alpha-positive, platinum-resistant ovarian, fallopian tube or peritoneal cancer^[10]^. The toxic payload is DM4, which we found to be a P-gp substrate in our screen. Testing MIRV with the KB3-1/KB-8-5-11 pair used in our screening assay and the OVCAR8/NCI-ADR-RES cell line pair, we found that the P-gp overexpressing lines KB 8-5-11 and NCI-ADR-RES were quite resistant to treatment with MIRV compared to the parental lines [Figure 3 and Supplementary Figure 2]. These results confirm that screening of isolated payloads for susceptibility to P-gp efflux translates to cellular resistance to an ADC carrying a P-gp-substrate toxin, and suggest that P-gp may contribute to clinical resistance to MIRV.

### Duocarmycins and PNU-159682 are highly toxic to a broad range of cancer cell lines

**Figure 4:**
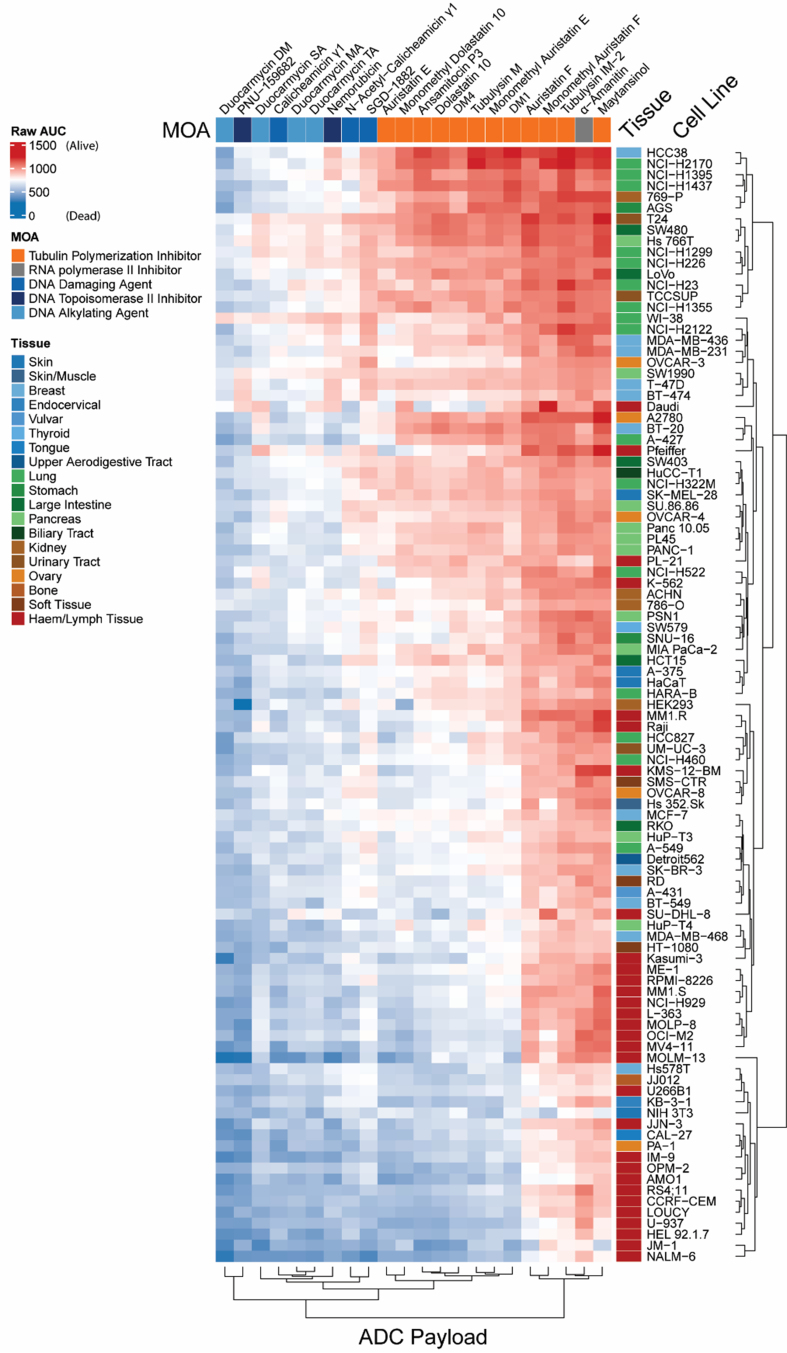
Differential cytotoxicity of ADC payloads across cancer cell lines from diverse tissue origins. Heatmap showing AUC values from dose–response viability assays for a panel of ADC payload compounds tested across a large set of human cancer cell lines. Each row represents a unique cancer cell line, annotated by tissue of origin (colored bar on the right-hand side), and each column corresponds to an ADC payload compound, annotated by mechanism of action on the top of the heatmap. AUC values are color-coded, with red indicating higher AUC (lower sensitivity; more resistant) and blue indicating lower AUC (higher sensitivity; more cytotoxic effect). Clustering (both rows and columns) was performed using hierarchical methods based on compound response profiles. ADC: Antibody-drug conjugate; AUC: area under the curve; MOA: mechanism of action.

We next explored the toxicity of the various payloads across a broad range of cell lines, including lines from cancers originating from the skin, kidney, pancreas, lung and blood. We excluded five payloads from our original list of 27 - vinblastine, SN-38, camptothecin, dasatinib and doxorubicin - as these compounds have been well characterized. As shown in Figure 4, hematologic cancers were sensitive to nearly all the compounds tested, while lung and pancreatic cancers tended to be less sensitive. The duocarmycins and PNU-159682 were uniformly toxic across the cell line panel. Given that the duocarmycins and PNU-159682 were not found to be substrates of ABC transporters, these compounds should be considered for use as ADC payloads in the future.

## DISCUSSION

We systematically screened 27 commonly used ADC payloads [Table 2] in two orthogonal, paired cell sets to identify substrates of the ABC transporters P-gp and ABCG2. Of the studied DNA-damaging agents, we identified calicheamicin analogs, a PBD dimer (SGD-1882), and doxorubicin to be substrates of the ABC transporter P-gp. Of the tubulin-targeting agents, vinblastine and all auristatin and maytansinoid derivatives were strong P-gp substrates, whereas MMAF was less susceptible to transport. SN-38 was identified as a substrate of ABCG2; however, no other cytotoxic payloads of the 27 payloads that were screened were found to be transported by ABCG2. In confirmatory studies with cells transfected with plasmids containing *ABCB1*, *ABCC1* or *ABCG2*, cells expressing P-gp were resistant to MMAE, DM1, DM4 and calicheamicin γ1. Cells expressing ABCG2 were resistant to the camptothecin derivatives exatecan and Dxd, while P-gp overexpression also conferred resistance to Dxd. Cells expressing P-gp, MRP1 or ABCG2 were resistant to all PBD dimers tested. Notably, none of the transporters conferred resistance to the duocarmycin analogs, camptothecin, nemorubicin, PNU-159682, and α-amanitin. Additionally, the duocarmycins, nemorubicin, and PNU-159682 were toxic to a wide range of cancer cell line models, suggesting their potential value for further development as ADC payloads.

Our results are in agreement with earlier *in vitro* studies using cell lines and *in vivo* mouse models which have demonstrated that overexpression of ABC transporters can drive resistance to some ADCs. Naito *et al.* found that leukemia cell lines that overexpressed P-gp were resistant to treatment with gemtuzumab ozogamicin and that combination with P-gp inhibitors such as valspodar or biricodar reversed resistance^[30]^. In the MRP1-overexpressing cell line HL-60/ADR, addition of the MRP1 inhibitor MK-571 was found to increase sensitivity to gemtuzumab ozogamicin, suggesting that MRP1 mediates resistance to the ADC^[31]^; however, overexpression of ABCG2 does not appear to cause resistance^[32]^. Studies using cell lines expressing P-gp, MRP1, or ABCG2 with the CD33-targeting ADC AVE9633 (payload DM4), showed that only P-gp could confer resistance^[21]^. Repeated treatment of mice xenografted with the SUM190 breast cancer cell line with the ADC N41mab-vcMMAE (payload MMAE)-induced refractory tumors which were found to overexpress P-gp as the mechanism of resistance^[33]^. An ADC targeting the delta-like non-canonical Notch ligand 1 protein (ADCT-701; payload SG3199) was found to be less effective in adrenocortical cancer cell lines and organoids with high expression of P-gp, and the free drug was also reported to be a substrate of P-gp^[34]^. The ADC OBI-992 (payload exatecan) was shown to retain efficacy in cellular models overexpressing P-gp, but ABCG2 conferred modest resistance^[35,36]^. Treatment of cells with both ADCs carrying SG3199 and SG3199 alone showed acquired resistance mediated by ABC transporters, with reversal of resistance by transporter siRNA or transporter inhibitors^[37]^. Notably, xenograft tumors with acquired resistance to MMAE-containing ADCs driven by P-gp overexpression were sensitive to the identical antibody-linker when PNU-159682 replaced MMAE as the payload^[38]^. The findings of these studies are consistent with our results, confirming that calicheamicin, DM4, MMAE, and SG3199 are substrates of P-gp and - given that PNU-159682 remains active in MMAE-resistant cells - suggesting that selecting payloads with low efflux potential may reduce the risk of acquired resistance.

Overexpression of P-gp has also emerged as a marker of resistance in a subset of patients who have been treated with gemtuzumab ozogamicin and brentuximab vedotin. Early studies examining leukemic blasts from patients with resistant disease demonstrated that P-gp and MRP1 expression could lead to resistance^[20,31,39]^. P-gp expression was found to inversely correlate with clinical response to gemtuzumab ozogamicin in patients with AML^[17]^. Similarly, a study examining a small cohort of lymphoma patients resistant to gemtuzumab vedotin reported P-gp overexpression in a patient with Hodgkin Lymphoma^[22]^. Finally, a case report of a patient with bladder cancer whose disease had progressed after treatment with enfortumab vedotin reported high levels of P-gp in the resistant tumor^[40]^.

These initial studies suggested that the addition of a P-gp inhibitor might be beneficial to counter acquired resistance via P-gp overexpression, leading to clinical trials combining P-gp inhibitors with ADC treatment. The P-gp inhibitor zosuquidar was found to reverse resistance to gemtuzumab ozogamycin in *ex vivo* studies using P-gp-positive blasts obtained from patients with resistant disease^[41]^. In a clinical trial combining zosuquidar with gemtuzumab ozogamicin, greater overall survival was noted in patients with P-gp-positive resistant disease^[42]^. A small clinical study combining cyclosporine with gemtuzumab ozogamicin in patients with resistant disease led to an increased overall and complete response rate^[43]^. However, the final findings from a larger trial (NCT03013933) exploring the combination of brentuximab vedotin with cyclosporine were less positive, largely due to toxicity from the use of cyclosporine A as the P-gp inhibitor, highlighting the importance of inhibitor choice when designing combination trials^[44,45]^. Beyond strategies for advancing future ADC development^[12]^, the addition of efflux inhibitors to overcome ADC resistance may offer opportunities to improve efficacy of currently approved ADCs.

We report duocarmycin analogs to be broadly cytotoxic and, in agreement with prior studies, not be substrates of P-gp or ABCG2, positioning them as improved ADC payloads^[46]^. Optimization of duocarmycin molecules has yielded prodrugs that can be activated upon release from an ADC [e.g., seco-duocarmycin-hydroxybenzamide-azaindole (seco-DUBA)]^[47]^, with promising preclinical reports on ADC efficacy^[48,49]^. Current clinical trials with Trastuzumab duocarmazine are testing the clinical efficacy of these developments to see if they yield less susceptibility to acquired resistance by payload efflux^[50]^.

Following localization to a target cell by the antibody, an ADC is internalized and the payload is released to exert its cytotoxic effect. Depending on the linker chemistry, the released species may retain a residual linker moiety with potential to alter its physicochemical properties and potential recognition by ABC transporters. For instance, DM1 alone is a substrate of P-gp, but DM1 released from Ado-trastuzumab emtansine is a charged metabolite, which may alter its efflux^[51]^. While our study focused on isolated ADC payload molecules and MIRV, which contains a cleavable linkage that yields free DM4 without any remaining linker moiety, prior studies have reported acquired resistance to ADCs with varied linker chemistries.

While our data strongly support a role for ABC transporters in mediating resistance to ADCs via payload efflux, we cannot exclude additional resistance mechanisms. Our experiments used a focused set of *in vitro* cellular systems and specific ADC/payload chemotypes, which may not fully capture the diversity of resistance that arises in patients; other processes reported in the literature, such as altered target antigen expression, changes in ADC trafficking or processing, or adaptations in DNA-damage and cell-death pathways, may also modulate ADC sensitivity. Finally, although we characterize ABC transporter-mediated payload efflux *in vitro*, future studies in appropriate *in vivo* models and with longitudinal clinical samples are needed to define how ABC transporters influence acquired resistance in the clinical setting.

In conclusion, we show that the payloads of several FDA-approved ADCs are strong substrates of P-gp, suggesting that active transport and efflux of the released payload may contribute to acquired resistance to clinical ADCs. These effects may also increase exposure of the cytotoxic payload to systemic tissues and could contribute to *in vivo* off-target toxicities. We identified ADC payloads that were not substrates of either P-gp or ABCG2 - notably the duocarmycin series and PNU-159682 - which were among the most broadly cytotoxic of all toxins tested in a panel of 99 cancer cell lines - and suggest that these compounds be prioritized as future ADC payloads due to their potential for reduced susceptibility to transporter-mediated acquired resistance^[52]^.

## DECLARATIONS

### Availability of data and materials

Data from the high-throughput screens has been submitted to the PubChem repository with AIDs 2061167 and 2202241. Data can be accessed via the links: https://pubchem.ncbi.nlm.nih.gov/bioassay/2061167 and https://pubchem.ncbi.nlm.nih.gov/bioassay/2202241.

### Copyright

© The Author(s) 2026.
