# Supplementary material for: Identification of antibody-drug conjugate payloads that are substrates of ATP-binding cassette drug efflux transporters
Source: Cancer Drug Resist. 2026 Jan 12;9:2. doi: 10.20517/cdr.2025.151 (PMC12883344; doi:10.20517/cdr.2025.151)
Supplement: Supplementary file 1 [file cdr-9-2-SupplementaryMaterials.pdf]

Supplementary Materials

Identification of antibody-drug conjugate payloads that are substrates of ATP-binding cassette drug efflux transporters

Jacob S. Roth[1], Hui Guo[1], Lu Chen[1], Min Shen[1], Omotola Gbadegesin[2], Robert W. Robey[2], Michael M. Gottesman[2], Matthew D. Hall[1]

[1]National Center for Advancing Translational Sciences, National Institutes of Health, Rockville, MD 20850, USA.
[2]Laboratory of Cell Biology, National Cancer Institute, National Institutes of Health, Bethesda, MD 20892, USA.

Correspondence to: Dr. Matthew D. Hall, National Center for Advancing Translational Sciences, National Institutes of Health, Rockville, MD 20850, USA.
E-mail: hallma@mail.nih.gov

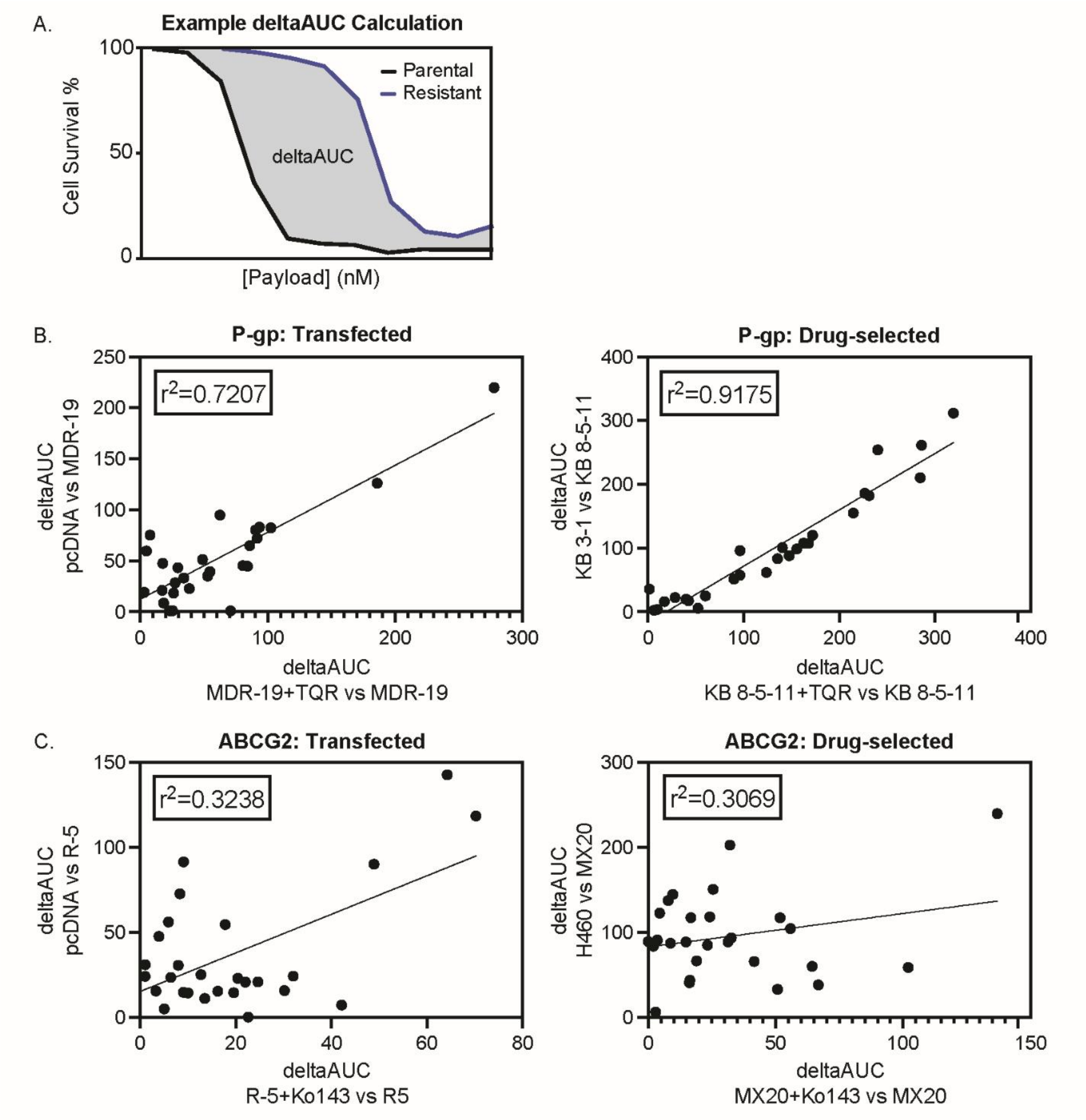

Supplementary Figure 1. DeltaAUC comparisons to validate efflux by P-gp and ABCG2. (A) Representative calculation of deltaAUC comparing compound activity between parental and resistant cell lines; (B) DeltaAUC comparisons for P-gp overexpression in transfected and drug-selected cell lines. Linear correlations confirm P-gp activity drives resistance, as the P-gp inhibitor tariquidar (TQR) sensitizes resistant cells; (C) DeltaAUC comparisons for ABCG2 overexpression in transfected and drug-selected cell lines. Poor linear correlations suggest that ABCG2 activity does not drive resistance observed for some molecules, as the ABCG2 inhibitor ko143 fails to re-sensitize resistant cells.

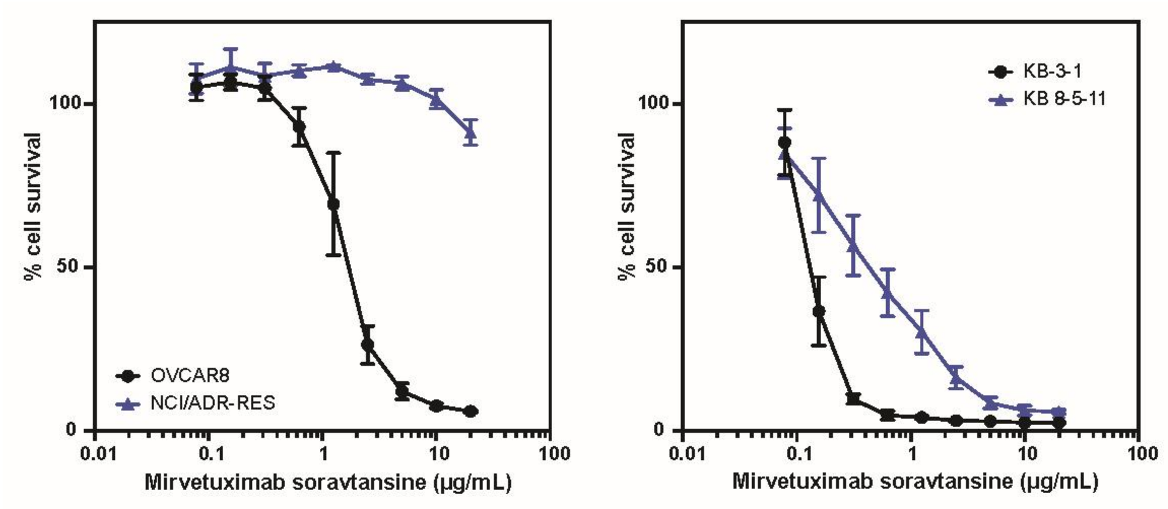

Supplementary Figure 2. Cytotoxicity assays with mirvetuximab soravtansine. Three-day cytotoxicity assays were performed with mirvetuximab soravtansine, as described in the Materials and Methods, using the OVCAR8/NCI-ADR-RES pair (left) and the KB 3-1/KB 8-5-11 pair (right). Results from the second of two independent experiments are shown.

Supplementary Table 1. Sourcing and growth conditions for cell lines used in Figure 4

Cell Name (Cellosaurus)	Tissue/Organ Origin	Media	Commercial Source	Catalog Number	Cellosaurus Accession Number
769-P	Kidney (Renal cell carcinoma)	RPMI +10% FBS	ATCC	CRL-1933	CVCL_1050
786-O	Kidney (Renal cell carcinoma)	RPMI +10% FBS	ATCC	CRL-1932	CVCL_1051
A-375	Skin (Melanoma)	RPMI +10% FBS	ATCC	CRL-1619	CVCL_0132
A-427	Lung (adenocarcinoma)	EMEM +10 % FBS	ATCC	HTB-53	CVCL_1055
A-431	Skin (Epidermoid carcinoma)	RPMI +10% FBS	ATCC	CRL-1555	CVCL_0037
A-549	Lung (Non-small cell lung carcinoma)	RPMI +10% FBS	ATCC	CCL-185	CVCL_0023
A2780	Ovary (Ovarian carcinoma)	RPMI +10% FBS	Cytion	300491	CVCL_0134
ACHN	Kidney (Renal cell carcinoma)	RPMI +10% FBS	ATCC	CRL-1611	CVCL_1067
AGS	Stomach (Gastric adenocarcinoma)	F12K +10 % FBS	ATCC	CRL-1739	CVCL_0139
AMO1	Blood (Multiple myeloma)	RPMI +20 % FBS	DSMZ	ACC 538	CVCL_1806
BT-20	Breast (Breast carcinoma)	EMEM +10 % FBS	ATCC	HTB-19	CVCL_0178
BT-474	Breast (Breast carcinoma)	RPMI +10% FBS	ATCC	HTB-20	CVCL_0179
BT-549	Breast (Breast carcinoma)	RPMI +10%	ATCC	HTB-122	CVCL_1092

		FBS			
CAL-27	Tongue (Squamous Cell Carcinoma)	DMEM +10 % FBS	ATCC	CRL-2095	CVCL_1107
CCRF-CEM	Blood (T-cell leukemia)	RPMI +10% FBS	ATCC	CCL-119	CVCL_0207
Daudi	Blood (Burkitt's lymphoma)	RPMI +10% FBS	ATCC	CCL-213	CVCL_0008
Detroit562	Head and Neck (Pharyngeal carcinoma)	EMEM +10 % FBS	ATCC	CCL-138	CVCL_1171
HaCaT	Skin (Human keratinocyte)	RPMI +10% FBS	Cytion	300493	CVCL_0038
HARA-B	Lung (Squamous cell carcinoma)	EMEM +10 % FBS	Cytion	300465	CVCL_2915
HCC38	Breast (Breast carcinoma)	RPMI +10% FBS	ATCC	CRL-2314	CVCL_1267
HCC827	Lung (adenocarcinoma)	RPMI +10% FBS	ATCC	CRL-2868	CVCL_2063
HCT15	Colon (Colorectal adenocarcinoma)	RPMI +10% FBS	ATCC	CCL-225	CVCL_0292
HEL 92.1.7	Blood (Erythroleukemia)	RPMI +10% FBS	ATCC	TIB-180	CVCL_2481
Hs 352.Sk	Skin (Non-cancerous)	RPMI +10% FBS	ATCC	CRL-7250	CVCL_N613
Hs 766T	Pancreas (Ductal carcinoma)	DMEM +10 % FBS	ATCC	HTB-134	CVCL_0334
Hs578T	Breast (Breast carcinoma)	RPMI +10% FBS	ATCC	HTB-126	CVCL_0332
HT-1080	Connective tissue (Fibrosarcoma)	RPMI +10% FBS	ATCC	CCL-121	CVCL_0317
HuCC-T1	Bile duct	RPMI	Riken	RCB1960	CVCL_03

	(Cholangiocarcinoma)	+10% FBS	Bioresource Center		24
HuP-T3	Pancreas (Adenocarcinoma)	RPMI +10% FBS	DSMZ	ACC 259	CVCL_1299
HuP-T4	Pancreas (Adenocarcinoma)	RPMI +10% FBS	DSMZ	ACC 223	CVCL_1300
IM-9	Blood (Multiple myeloma)	RPMI +10% FBS	ATCC	CCL-159	CVCL_1305
JJ012	Bone (Chondrosarcoma)	RPMI +10% FBS	Gift	Karina Galoian (University of Miami)	CVCL_D605
JJN-3	Blood (Multiple myeloma)	IMDM +20 % FBS	DSMZ	ACC 541	CVCL_2078
JM-1	Blood (B-cell Lymphoma)	RPMI +10% FBS	ATCC	CRL-10423	CVCL_3532
K-562	Blood (Chronic myelogenous leukemia)	RPMI +10% FBS	ATCC	CCL-243	CVCL_0004
Kasumi-3	Blood (Acute myeloid leukemia)	RPMI +10% FBS	ATCC	CRL-2725	CVCL_0612
KMS-12-BM	Blood (Multiple myeloma)	RPMI +10% FBS	DSMZ	ACC 551	CVCL_1334
L-363	Blood (Multiple myeloma)	RPMI+20 % FBS	DSMZ	ACC 49	CVCL_1357
LOUCY	Blood (T-cell leukemia)	RPMI +10% FBS	ATCC	CRL-2629	CVCL_1380
LoVo	Colon (Colorectal adenocarcinoma)	RPMI +10% FBS	ATCC	CCL-229	CVCL_0399
MCF-7	Breast (Breast adenocarcinoma)	RPMI +10% FBS	ATCC	HTB-22	CVCL_0031
MDA-MB-231	Breast (Breast carcinoma)	RPMI +10% FBS	ATCC	HTB-26	CVCL_0062

MDA-MB-436	Breast (Breast carcinoma)	RPMI +10% FBS	ATCC	HTB-130	CVCL_0623
MDA-MB-468	Breast (Breast carcinoma)	RPMI +10% FBS	ATCC	HTB-132	CVCL_0419
ME-1	Blood (Acute Myeloid Leukemia)	RPMI + 20 % FBS	ATCC	HB-119	CVCL_M535
MIA PaCa-2	Pancreas (Ductal carcinoma)	RPMI +10% FBS	ATCC	CRL-1420	CVCL_0428
MM1.R	Blood (Multiple myeloma)	RPMI +10% FBS	ATCC	CRL-2975	CVCL_8794
MM1.S	Blood (Multiple myeloma)	RPMI +10% FBS	ATCC	CRL-2974	CVCL_8792
MOLM-13	Blood (Acute myeloid leukemia)	RPMI + 20 % FBS	DSMZ	ACC 554	CVCL_2119
MOLP-8	Blood (Multiple myeloma)	RPMI +20 % FBS	DSMZ	ACC 569	CVCL_2124
MV4-11	Blood (Acute myeloid leukemia)	RPMI +20 % FBS	ATCC	CRL-9591	CVCL_0064
NALM-6	Blood (Acute lymphoblastic leukemia)	RPMI +10% FBS	ATCC	CRL-3273	CVCL_0092
NCI-H1299	Lung (Non-small cell lung carcinoma)	RPMI +10% FBS	ATCC	CRL-5803	CVCL_0060
NCI-H1355	Lung (adenocarcinoma)	RPMI +10% FBS	ATCC	CRL-5865	CVCL_1464
NCI-H1395	Lung (adenocarcinoma)	RPMI +10% FBS	ATCC	CRL-5868	CVCL_1467
NCI-H1437	Lung (adenocarcinoma)	RPMI +10% FBS	ATCC	CRL-5872	CVCL_1472
NCI-H2122	Lung (adenocarcinoma)	RPMI +10%	ATCC	CRL-5985	CVCL_1531

		FBS			
NCI-H2170	Lung (Squamous cell carcinoma)	RPMI +10% FBS	ATCC	CRL-5928	CVCL_1535
NCI-H226	Lung (Squamous cell carcinoma)	RPMI +10% FBS	ATCC	CRL-5826	CVCL_1544
NCI-H23	Lung (Non-small cell lung carcinoma)	RPMI +10% FBS	ATCC	CRL-5800	CVCL_1547
NCI-H322M	Lung (adenocarcinoma)	RPMI +10% FBS	NIH	NCI-60	CVC_1557
NCI-H460	Lung (Large cell carcinoma)	RPMI +10% FBS	ATCC	HTB-177	CVCL_0459
NCI-H522	Lung (Non-small cell lung carcinoma)	RPMI +10% FBS	ATCC	CRL-5810	CVCL_1567
NCI-H929	Blood (Multiple myeloma)	RPMI +10% FBS	ATCC	CRL-3580	CVCL_1600
NIH 3T3	Skin (Mouse fibroblast)	RPMI +10% FBS	ATCC	CRL-1658	CVCL_0594
OCI-M2	Blood (Acute myeloid leukemia)	RPMI +10% FBS	DSMZ	ACC 619	CVCL_2150
OPM-2	Blood (Multiple myeloma)	RPMI +10% FBS	DSMZ	ACCC 50	CVCL_1625
OVCAR-3	Ovary (Ovarian carcinoma)	RPMI +10% FBS	ATCC	HTB-161	CVCL_0465
OVCAR-4	Ovary (Ovarian carcinoma)	RPMI +10% FBS	Gift	Phil Lorenzi MD Anderson	CVCL_1627
OVCAR-8	Ovary (Ovarian carcinoma)	RPMI +10% FBS	Gift	Phil Lorenzi MD Anderson	CVCL_1629
PA-1	Ovary (Ovarian teratocarcinoma)	EMEM +10 % FBS	ATCC	CRL-1572	CVCL_0479
Panc 10.05	Pancreas (Ductal	RPMI	ATCC	CRL-2547	CVCL_16

	carcinoma)	+10% FBS			39
PANC-1	Pancreas (Ductal carcinoma)	RPMI +10% FBS	ATCC	CRL-1469	CVCL_0480
Pfeiffer	Blood (Diffuse large B-cell lymphoma)	RPMI +10% FBS	ATCC	CRL-2632	CVCL_3326
PL-21	Blood (Acute myeloid leukemia)	RPMI +20 % FBS	DSMZ	ACC 536	CVCL_2161
PL45	Pancreas (Ductal carcinoma)	RPMI +10% FBS	ATCC	CRL-2558	CVCL_3567
PSN1	Pancreas (Ductal carcinoma)	RPMI +10% FBS	ATCC	CRL-3211	CVCL_1644
Raji	Blood (Burkitt's lymphoma)	RPMI +10% FBS	ATCC	CCL-86	CVCL_0511
RD	Muscle (Rhabdomyosarcoma)	RPMI +10% FBS	Gift	Mari Yohe NCI	CVCL_1649
RKO	Colon (Colorectal adenocarcinoma)	EMEM + 10 % FBS	ATCC	CRL-2577	CVCL_0504
RPMI-8226	Blood (Multiple myeloma)	RPMI +10% FBS	ATCC	CRM-CCL-155	CVCL_0014
RS4;11	Blood (Acute lymphoblastic leukemia)	RPMI +10% FBS	ATCC	CRL-1873	CVCL_0093
SK-BR-3	Breast (Breast carcinoma)	RPMI +10% FBS	ATCC	HTB-30	CVCL_0033
SK-MEL-28	Skin (Melanoma)	RPMI +10% FBS	ATCC	HTB-72	CVCL_0526
SMS-CTR	Muscle (Rhabdomyosarcoma)	RPMI +10% FBS	Gift	Mari Yohe NCI	CVCL_A770
SNU-16	Stomach (Gastric carcinoma)	RPMI +10% FBS	ATCC	CRL-5974	CVCL_0076
SU-DHL-8	Blood (Diffuse	RPMI	ATCC	CRL-2961	CVCL_22

	large B-cell lymphoma)	+10% FBS			07
SU.86.86	Pancreas (Ductal carcinoma)	RPMI +10% FBS	ATCC	CRL-1837	CVCL_3881
SW1990	Pancreas (Ductal carcinoma)	RPMI +10% FBS	ATCC	CRL-2172	CVCL_1723
SW403	Colon (Colorectal adenocarcinoma)	RPMI +10% FBS	ATCC	CCL-230	CVCL_0545
SW480	Colon (Colorectal adenocarcinoma)	RPMI +10% FBS	ATCC	CCL-228	CVCL_0546
SW579	Thyroid (Thyroid carcinoma)	RPMI +10% FBS	ATCC	HTB-107	CVCL_3603
T-47D	Breast (Breast carcinoma)	RPMI +10% FBS	ATCC	HTB-133	CVCL_0553
T24	Bladder (Transitional cell carcinoma)	RPMI +10% FBS	ATCC	HTB-4	CVCL_0554
TCCSUP	Bladder (Transitional cell carcinoma)	EMEM +10 % FBS	ATCC	HTB-53	CVCL_1738
U-937	Blood (Histiocytic lymphoma)	RPMI +10% FBS	ATCC	CRL-1593.2	CVCL_0007
U266B1	Blood (Multiple myeloma)	RPMI +15% FBS	ATCC	TIB-196	CVCL_0566
UM-UC-3	Bladder (Urothelial carcinoma)	EMEM +10 % FBS	ATCC	CRL-1749	CVCL_1783
WI-38	Lung (Human lung fibroblast)	EMEM +10 % FBS	ATCC	CCL-75	CVCL_0579

Suppliers: ATCC, Manassas VA; DSMZ, Braunschweig-Süd,Germany; Riken Bioresource Center, Tsukuba, Ibaraki, Japan; Cytion, Sioux Falls, SD; NIH, DCDT Tumor Repository, National Cancer Institute at Frederick, MD.
